# Biomechanical evaluation of hybrid double plate osteosynthesis using a locking plate and an inverted third tubular plate for the treatment of proximal humeral fractures

**DOI:** 10.1371/journal.pone.0206349

**Published:** 2018-10-29

**Authors:** Jan Theopold, Stefan Schleifenbaum, Mirijam Müller, Michael Werner, Niels Hammer, Christoph Josten, Pierre Hepp

**Affiliations:** 1 Department of Orthopedic, Trauma and Plastic Surgery, University of Leipzig, Leipzig, Germany; 2 ZESBO–Zentrum zur Erforschung der Stuetz- und Bewegungsorgane, Leipzig, Germany; 3 Fraunhofer Institute for Machine Tools and Forming Technology, Dresden, Germany; 4 Department of Anatomy, University of Otago, Dunedin, New Zealand; Politecnico di Milano, ITALY

## Abstract

**Background:**

Treating proximal humerus fractures can be challenging because of large metaphyseal defects that conceal anatomical landmarks. In such cases, medial cortical support with, for example, calcar screws, is mandatory. Nevertheless, varus dislocations and implant failures in patients with impaired bone quality persist. Thus, the need for effective treatment of these patients exists. Hybrid double plate osteosynthesis was introduced as an alternative, yielding similar results as calcar screws. However, a biomechanical comparison of the stability of these two techniques is pending.

**Methods:**

Cadaveric humeral specimens were treated with plate osteosynthesis and calcar screws (group 1, n = 9) or hybrid double plate osteosynthesis (group 2, n = 9) using a proximal humerus fracture model with a two-part fracture. Displacement, stiffness, failure mode, and ultimate load were examined biomechanically in a cyclic compressive-loading scenario.

**Results:**

Although the hybrid double plate osteosynthesis (group 2) tended to confer higher stiffnesses than the medial support screws at higher cycles (group 1), this trend was below the level of significance. The displacement revealed non-significantly lower values for group 1 as compared with group 2 for cycles 50 and 2000, but at 5000 cycles, group 2 offered non-significantly lower displacement values than group 1. The ultimate load tended to be non-significantly higher in the hybrid double plate osteosynthesis group (group 2: 1342±369 N, group 1: 855±408 N). Both groups yielded similar failure rates, with the majority of failures in group 2 being gap closures (n = 8), whereas those in group 1 being plate dislocations (n = 4).

**Conclusions:**

The use of an additive plate osteosynthesis in the region of the bicipital groove may be a potential alternative to the previously-established method of using calcar screws. The biomechanical data obtained in this study suggests that hybrid double plate osteosynthesis is as rigid and robust as calcar screws.

## Introduction

Complex proximal humerus fractures are challenging to treat surgically because of concerns involving both anatomical reduction and the stability of osteosynthesis [1,2]. These fractures might extend metaphyseally or even conceal anatomical landmarks, thereby impairing the possibility for anatomical reduction. Moreover, the missing metaphyseal substance may limit the opportunity for a biomechanically stable surgical solution. A missing restoration of the medial cortical support has been identified as a factor responsible for increased failure rates [1,2] and non-unions [3] of the proximal humerus. This missing support may cause secondary varus dislocation with decreased stability because of supraspinatus deficiency [4]. To increase medial support, locking plates have been described in combination with fibular bone grafts or calcar screws [1,5]. Related problems with bone grafts such like impaction and harvesting [5,6] were reported. Although calcar screws may be considered a safe additive osteosynthesis, cases of implant failure have been reported frequently [7]. Thus, a more stable form of osteosynthesis and the use of hybrid double plating have been considered in single cases to enhance stability [8–12]. Double-plate osteosynthesis with one laterally and a second ventrally-placed third tube plate has proven to provide enhanced stability compared to other osteosyntheses, especially under torsion [13]. The use of two plates arranged at an angle of 90-degrees has been shown to be particularly effective for treating the distal humerus surgically [10]. Bai et al. report that calcar screws increase the axial and shear stiffness of the humerus but fail to enhance the overall biomechanical stability. Hence, another medial support could be an effective strategy [14]. Choi et al. developed a double-plate fixation technique for multi-fragmentary proximal humeral fractures using a polyaxial locking distal radius plate to prevent mal- and non-union or varus-shaped collapse of the head-shaft part by severe destruction of the medial support [12]. Theoretically, the combined use of a medial plate and a second lateral locking plate would directly provide a strong 2-column stability [15]. Previously, combinations of a mono-axial locking plate with an inverted third-tubular plate were implanted to fulfill this purpose, yielding a Constant score of 80 points at the one-year follow-up, indicating good shoulder function [16]. However, a biomechanical comparison of the stability of calcar screws and double plating is still pending. The clinical implications of this biomechanical study largely relate to optimizing the treatment of proximal humerus fractures. In the given study, human cadaveric specimens were treated with calcar screws (group 1) or double-plate osteosynthesis (group 2), using a proximal humerus injury model with a two-part fracture as described previously [17–21]. The following hypotheses were investigated:

1. Hybrid double-plate osteosynthesis displays higher stiffness and lower displacement than calcar screws for the treatment of a two-part fracture of the proximal humerus than calcar screws only.
2. Implant failure rates are lower following hybrid double-plate osteosynthesis than they are following the use of calcar screws for the treatment of a two-part fracture of the proximal humerus.
3. The failure load is higher for hybrid double-plate osteosynthesis as compared with calcar screws for the treatment of a two-part fracture of the proximal humerus.

## Materials and methods

### Specimens

**Fig 1:**
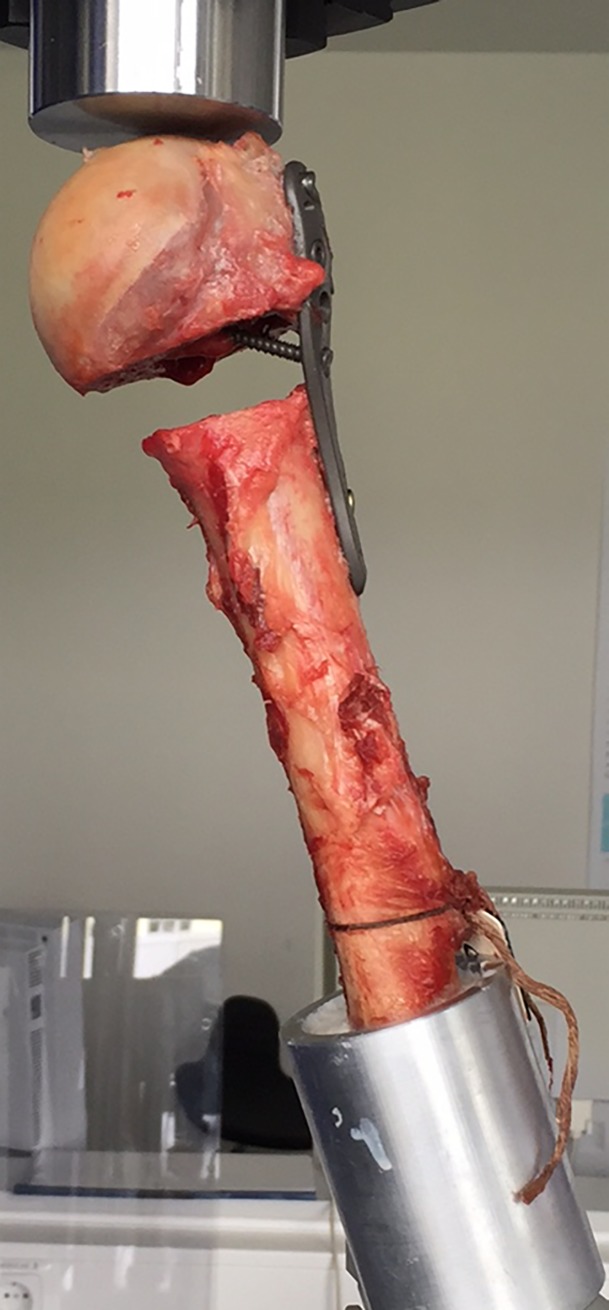
Structure of the experimental setup for the cycling test.

Twenty-two humerus specimens from 11 human cadavers (seven males, four females) were obtained bilaterally in a fresh and anatomically unfixed condition. The mean age was 66.8 ± 17.1 years. While alive all body donors gave their informed and written consent to the donation of their bodies for teaching and research purposes. Being part of the body donor program regulated by the Saxonian Death and Funeral Act of 1994 (third section, paragraph 18 item 8), institutional approval for the use of the post-mortem tissues of human body donors was obtained from the Institute of Anatomy, University of Leipzig. The authors declare that all experiments were conducted according to the principles of the Declaration of Helsinki. The specimens were stored at −83°C and the anteroposterior views were X-rayed before the tests. Macroscopically and radiographically, no signs of osteoarthritis or focal bone disease were visible. Seven pairs (bilateral) and four individual humeri were obtained. A dual energy X-ray absorptiometry (DXA) analysis was performed to determine bone mineral density (Hologic Delphi A QDR Series, Bedford, MA, USA). Prior to the mechanical tests, the humeri were osteotomized distally to a standard length of 220 mm. The osteotomized end was inserted into an aluminum cylinder and fixed using the polyurethane resin RENCAST FC52 Isocyanate/FC52 Polyol/Ceramic Powder (RenShape Solutions, Huntsman International LLC, Salt Lake City, UT, USA) of ratio 1/1/4 (Fig 1). The humeri were then divided into the two groups, ensuring that the same number of left and right humeri were present in each group to minimize the impact of interindividual differences in bone quality. The individual ones were assigned randomly.

### Implants

**Fig 2:**
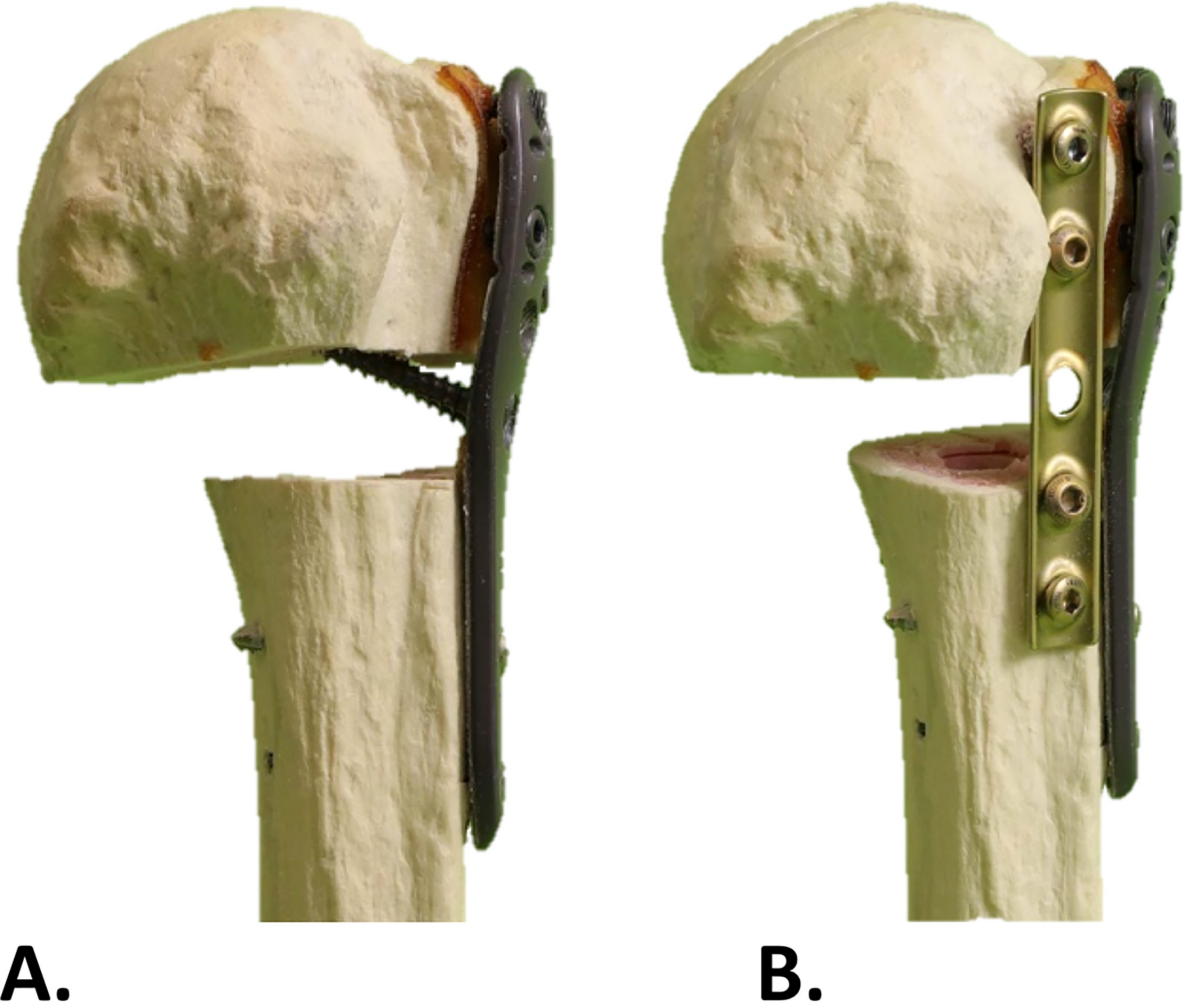
A. Group 1: supplied with a locking plate, five locking screws and two additional screws for medial support B. Group 2: supplied with the same locking plate setup as group 1 with an inverted five-hole one-third tube plate placed in the bicipital sulcus.

Each humerus was treated surgically with a locking plate (WinstaPH, Fa. Axomed, Freiburg i. Br., Germany) and five locking screws in accordance with the implant manufacturer’s specifications for use in the area of the greater tubercle. The screw length was determined individually for each hole by an experienced surgeon (JT). Subsequently, a transverse osteotomy was performed with an oscillating saw. The osteotomy was standardized with a fracture gap of 10 mm below the humeral neck. Group 1 was operated on with two additional medial support screws in accordance with the studies of Katthagen et al. and Zhang et al. (Fig 2A) [18,21]. Group 2 was treated using an additional plate osteosynthesis (Fig 2B). In this case, an inverted five-hole standard AO, one-third tube plate (KFI, Depuy-Synthes, Zuchwil, Switzerland) placed in the bicipital sulcus was used. The one-third tube plate was fixed with four standard AO cortical screws of corresponding length.

### Mechanical testing

A cyclic compressive loading scenario with the humerus in a 20-degree abduction position was performed, similar to the investigations of Katthagen et al. [18]. The force was applied in a sinusoidal form (1 Hz), ranging from a minimum of 10 N to a maximum of 250 N for 5000 cycles [14,17,18,22]. In the tests, the humerus were treated with normal saline every 10 minutes to hydrate them. During the tests, the force and displacement were measured using a servo-hydraulic uniaxial testing machine (Dynamess, DYNA-MESS Prüfmaschinen GmbH, Aachen, Germany). Subsequently, a failure test with 0 degrees of abduction of the humerus and a testing speed of 0.1 mm per second was performed [17,18,22,23]. The following failure criteria were defined: a) gap closure, b) fracture of the humeral head, or c) shaft, or d) implant failure.

For the cyclic tests, the system stiffness and relative stiffness (with the first cycle set to 100%) were calculated as a quotient of force and the displacement at 50, 2000 and 5000 cycles. For the same cycles, the displacement and relative displacement (with the first cycle set to 100%) were determined. During the failure tests, the strain and related displacements were investigated.

### Statistical analyses

The data were compared descriptively using Excel 2013 (Microsoft Corporation, Redmond, WA, USA). In addition, the data were examined statistically for Gaussian distribution, which was followed by the performance of a Mann-Whitney U-test or Student’s t-test for independent samples utilizing SPSS 24.0 (IBM, Armonk, NY, USA). The level of statistical significance was set at p < 0.05.

## Results

### Specimens

On DXA, the specimens showed a mean bone mineral density (BMD) of 0.57±0.14 g/cm^2^ for group 1 and 0.49±0.12 g/cm^2^ for group 2, being similar and non-significantly different (p = 0.156; Table 1).

**Table 1:** Baseline data of the body donors.

Cadaver ID	Age [years]	Gender	Body weight [kg]	Group 1 Bone mineral density [g/cm^2^]	Group 2 Bone mineral density [g/cm^2^]
1	92	female	37	0.47	0.41
3	65	female	98	0.73	0.72
11	87	female	62	0.50	0.47
12	78	male	56	0.52	
13	83	male	67		0.54
24	65	female	59	0.67	
35	90	female	84	0.60	0.42
48	82	male	87	0.53	0.62
49	89	male	64	0.33	0.45
70	76	male	98		0.45
118	93	female	54	0.78	0.32

### Cyclic mechanical testing

**Fig 3:**
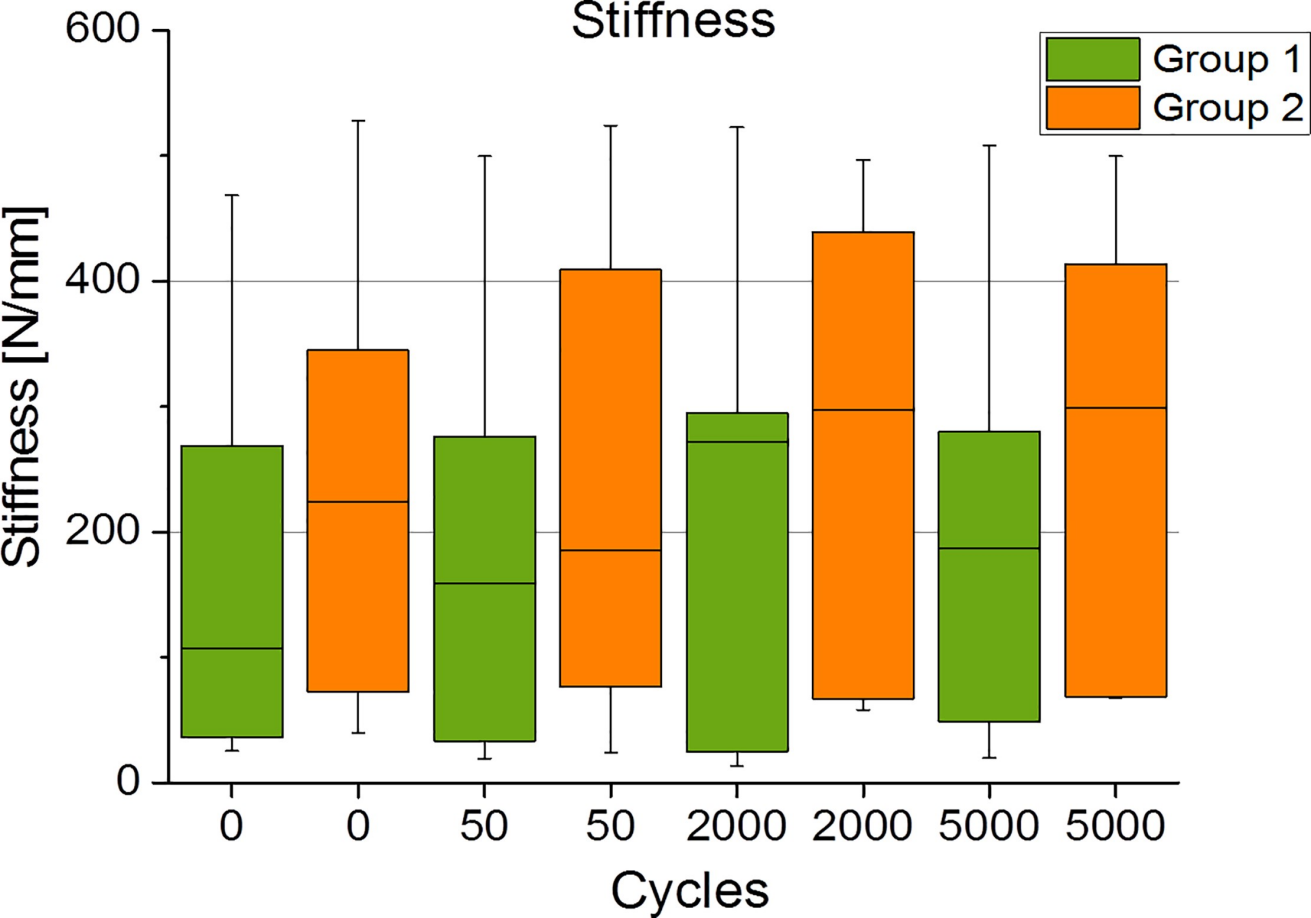
Boxplot of the stiffness after 0, 50, 2000 and 5000 cycles.

All humerus specimens were tested cyclically for 5000 cycles each, without showing any signs of preliminary failure. No macroscopically visible loosening of the cement/bracket was observed throughout the study. The mechanical tests yielded non-significant differences regarding stiffness and relative stiffness for 50 cycles (group 1: 207±181 N/mm; 102±20% vs. group 2: 272±172 N/mm; 141±69%); 2000 cycles (group 1: 231±205 N/mm; 114±68% vs. group 2: 324±283 N/mm; 154±143%); and 5000 cycles (group 1: 226±199 N/mm; 126±147% vs. group 2: 237±164 N/mm; 158±142%) (Fig 3). There were also no significant differences between the groups with respect to the relative displacements because of repeated loading. After 50 and 2000 cycles, the displacement was lower in group 1 (22±10%; 60±26%) than in group 2 (27±23%; 73±32%). After 5000 cycles, this ratio inverted so that higher displacements and variations were observed in group 1 (108±107%) as compared with group 2 (84±36%).

### Failure tests

**Fig 4:**
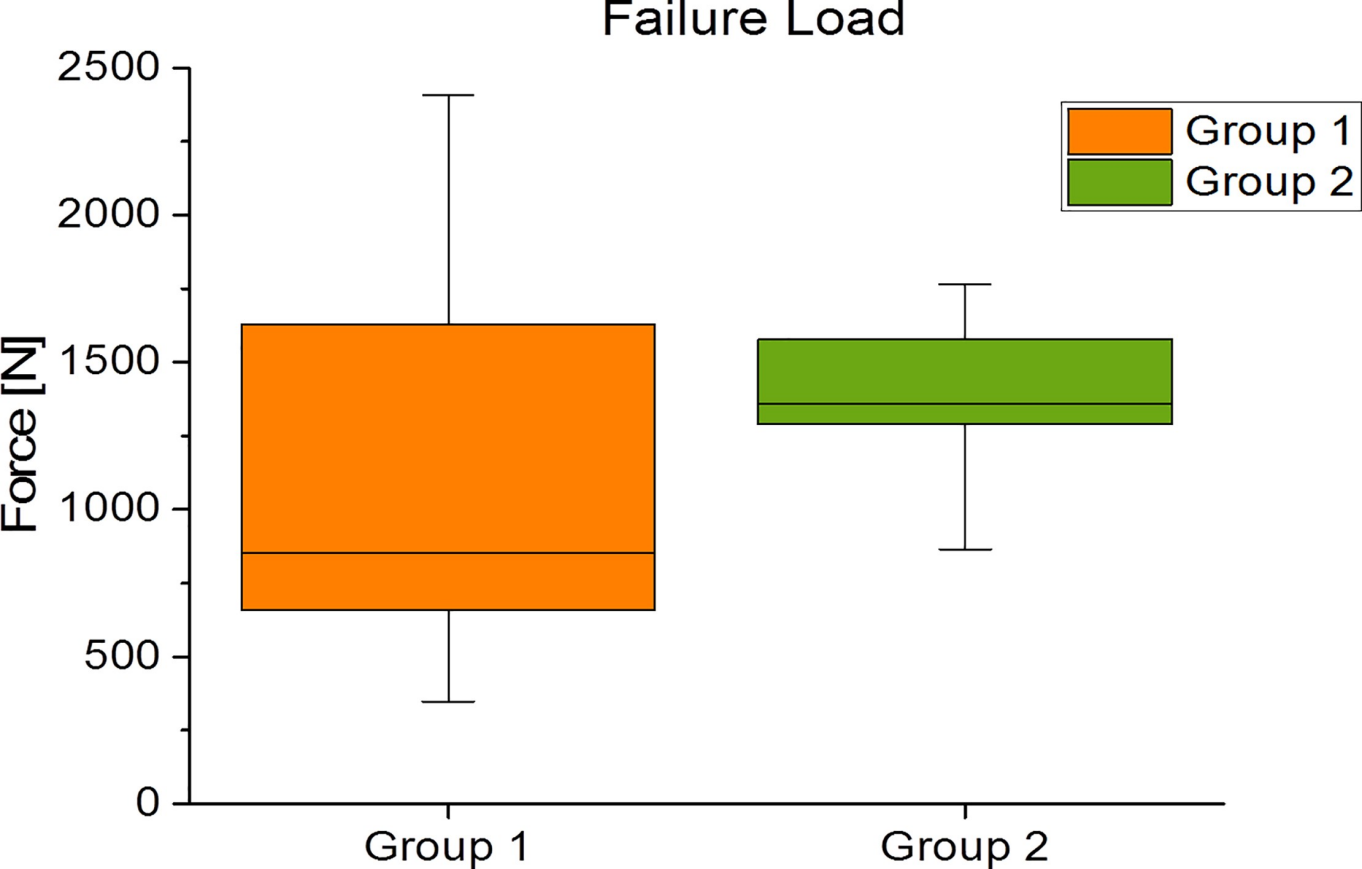
Boxplot of the breaking load.

In group 1, five gap closures and four plate dislocations were observed following fracturing of the humeral shaft (failure criteria a and c), while group 2 demonstrated eight gap closures and one plate dislocation (failure criteria a and c). Group 2 showed a non-significant higher failure load of 1342±369 N than group 1 with 855±408 N (p = 0.382) (Fig 4).

## Discussion

Complex proximal humerus fractures, which extend metaphyseally, impair the possibility for anatomical reduction and also a biomechanically-stable fixation. The medial cortical support is a hallmark of withstanding regarding implant failure [1,2], non-unions [3], secondary varus dislocation and related decreased stability due to reduced supraspinatus efficiency [4].

Additive calcar screw osteosynthesis has been described to reduce these complications, but it has little impact on implant failure and secondary dislocations [5–7,18,24,25]. Cement augmentation might be another measure to enhance the stability in the osteoporotic bone [7,26]. However, given that fracture lines might be directed into the joint cavity especially in multi-fragmentary proximal humeral fractures, augmentation poses the risk of bone cement flowing into the joint cavity. Hence, other additive procedures using additional plate osteosynthesis were investigated, yielding favorable results in cadaver tests, finite element analyses, and clinical trials [8,9]. Nonetheless, comparative studies investigating the biomechanical superiority of this procedure are still rare.

The focus of the given paper was set on the biomechanical evaluation of a hybrid plating technique compared to standard plating, using the gold standard of mechanical testing with unembalmed (fresh) human tissues. The findings give a certain direction towards the clinical applicability of the method, and will form the basis for future clinical studies on the topic.

The present study compared the biomechanical properties of plate osteosynthesis and additional screw support to the hybrid double plate osteosynthesis, which is comprised of a locking plate and inverted third tubular plate placed in the bicipital groove. Our biomechanical approach shown here specifically addressed the two-part model of such injury instead of the three- or four-part model, with the latter being biomechanically more complex and less feasible to standardize. The two-part model shown here has been established and widely used to answer biomechanical questions concerning the proximal humerus [16,21,22,27]. For this purpose, human cadaveric specimens with an average age of 66.8 ± 17.1 years and BMD values corresponding to the patients who typically suffer from proximal humerus fractures were used [28,29]. Mean BMD was non-significantly lower in the group with the hybrid plating technique than in the reference group.

Although the hybrid osteosynthesis group (group 2) showed a higher stiffness than the medial support screws group (group 1) for all investigated cycles, no level of significance was achieved. Investigating the displacement revealed a non-significantly lower displacement for cycles 50 and 2000 for group 1 as compared with group 2. In contrast, at 5000 cycles, group 2 offered non-significantly lower displacement values than group 1. Thus, we have to reject our hypothesis that hybrid double-plate osteosynthesis displays higher stiffness and lower displacement than calcar screws for the treatment of a two-part fracture of the proximal humerus. Nonetheless, caution must be taken in interpreting our finding and further studies are required.

**Fig 5:**
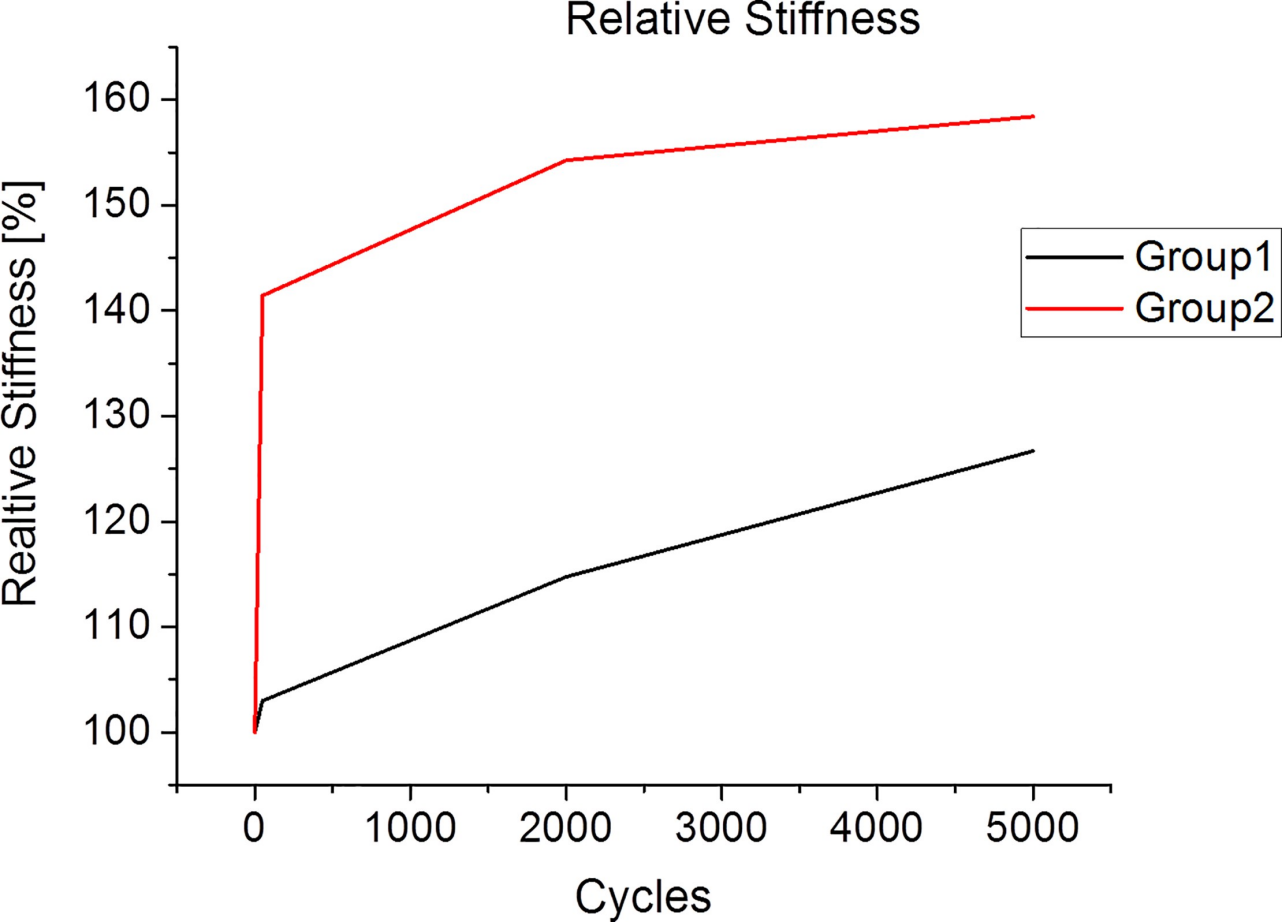
The curve of the average relative stiffness of the cyclic test for each group and the first cycle set to 100%.

Interestingly, between 2000 and 5000 cycles in group 1, the relative displacement abruptly increased, possibly representing a loss of the screw fixation. Although, again, a level of significance was not achieved here, we still assume a trend toward a higher stiffness for group 2 exists based on the analyses of the relative displacement over the investigated cycles (Fig 5). Considering these results, group 2 seemed to be more rigid during the loading, whereas the initial stiffness tended to be lower than in group 1. Thus, in line with the results of Lill et al., the hybrid double plate osteosynthesis might give preferable results [30]. Especially for young patients with good bone substance and in cases of highly complex multi-fragment fractures, which were previously examined using hybrid double osteosynthesis, more rigid implants should be used [17,30].

Moreover, in the failure tests, both groups yielded similar failure rates. However, the majority of the failures in group 2 were gap closures [8], whereas group 1 displayed more plate dislocations [9]. Similar to the study by Zhang et al., our study demonstrated a difference in the failure pattern [21]. Double plate osteosynthesis does not seem to adequately prevent a varus tilt of the humerus under great force. However, the changed power dissipation appears to shift in favor of the torsion, which at least prevents the screws from cutting through the head, similar to the medial support screws. Here, again, at least the rate of screw perforations was reduced in the two-part model [31]. Although we have to reject our hypothesis of a decrease in implant failure being associated with the use of hybrid double-plate osteosynthesis rather than with the use of calcar screws for the treatment of a two-part fracture of the proximal humerus, the failure events might differ in other cases, implicating an altered load distribution.

Moreover, failure loads of hybrid double-plate osteosynthesis as compared with calcar screws for the treatment of a two-part fracture of the proximal humerus displayed non-significant differences, contradicting our third hypothesis. The findings for the similar (non-significantly higher) ultimate load values in the hybrid osteosynthesis may indicate that a limited fracture gap might cause the absence of significant differences. This does reduce the inherent elasticity of the bone and cannot be ruled out as a source of failure. Because of different biomechanical setups, an adequate comparison between our data and the biomechanical data obtained by other groups is limited. However, as both our samples were evaluated using the same method, an internal comparison of the groups is possible, whereas a comparison of the absolute values with those of other working groups is only possible to a limited extent [21,22].

### Limitations

The effects of water content, drying, autolysis and freezing, cannot completely excluded and thus limit the estimation of a realistic behavior [32]. The number of shoulders examined is also limited. However, most biomechanical studies on this topic have similar numbers of cases [14,18,21,30]. Furthermore, solely tissue samples of elderly patients were examined limiting the translation of the present data treating younger patients. Related to the artificial setup of a biomechanical study, the physiological conditions including muscle forces and fracture healing are not represented [33]. In particular, the varus tension of the rotator cuff is not considered. Though the simplified two-part model of the fracture provides good reproducibility, more complex fractures are not taken into account [5,19,33]. Here it should be that those fracture would be even more the indication for the presented surgical treatment.

## Conclusions

Considering the presented results additive plate osteosynthesis in the region of the bicipital groove might be a possible alternative to the previously established medial support screw method treating proximal humeral fractures in a clinical environment although biomechanically there is no significant difference. Use of this hybrid osteosynthesis leads to similar displacement and stiffness and decreased plate dislocations, as the use of medial support screws. Furthermore, as compared with the medial support screws, hybrid osteosynthesis displayed a higher ultimate load before failure. Concerning the failure mode, the hybrid double plating technique appears to alter load distribution, fulfilling the additional benefits provided by medial support screws to prevent a cutting out of the screws in the humeral head. The key advantage of the hybrid plating system in the bicipital groove provides a potential alternative to the medial support screw method, changing load-deformation and failure in a similar manner as medial support screws. Furthermore, with the use of this implant construct, the events of plate dislocations might be reduced even though the varus tilt might be not prevented.

## Supporting information

S1 Table Table with the raw data. (XLSX)
